# Supplementary figures and images for: Whole exome sequencing of ENU-induced thrombosis modifier mutations in the mouse
Source: PLoS Genet. 2018 Sep 6;14(9):e1007658. doi: 10.1371/journal.pgen.1007658 (PMC6143275; doi:10.1371/journal.pgen.1007658)

A

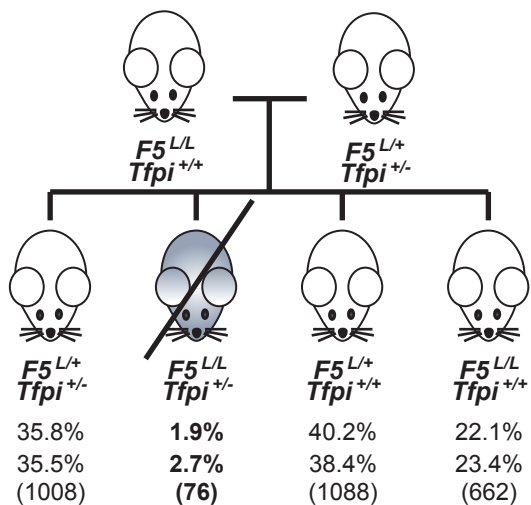

B

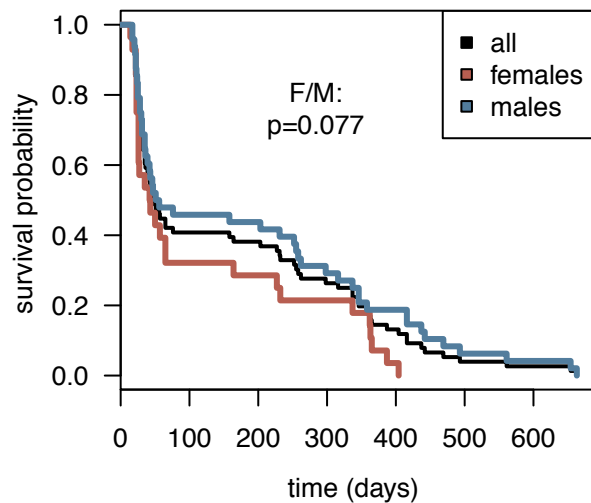

C

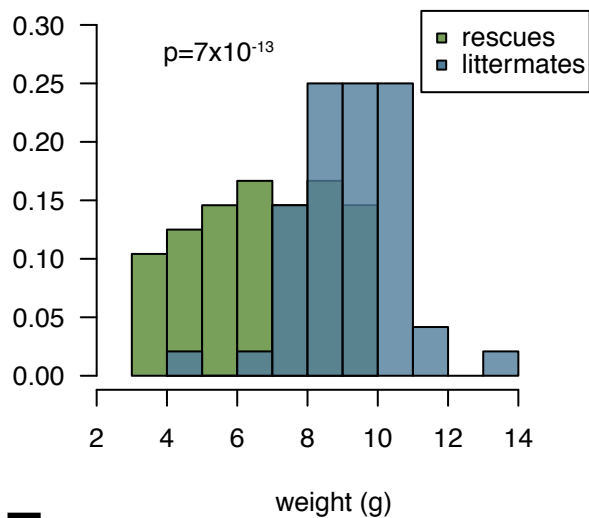

D

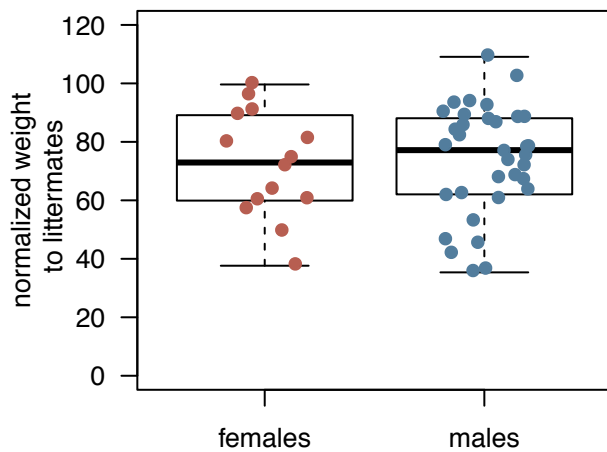

E

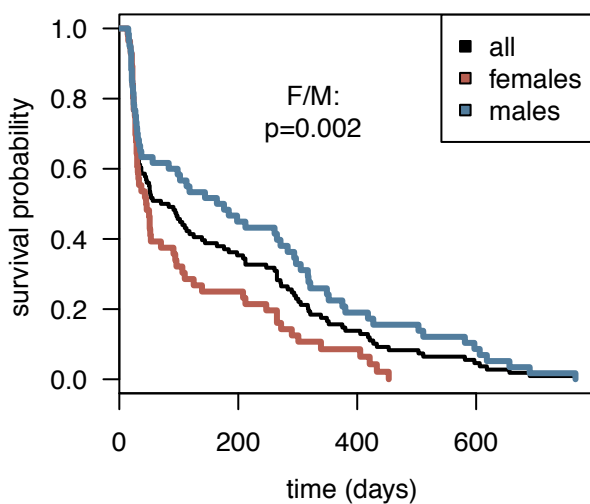

F

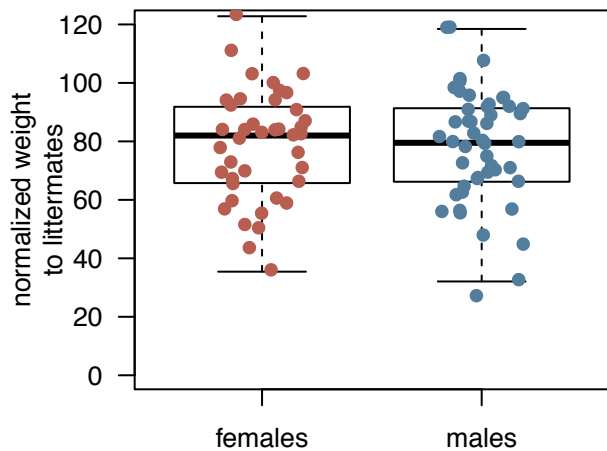

Supplement: S1 Fig — A) The ENU screen strategy is depicted here, along with the total numbers of G1 offspring observed by genotype. B) Survival curves for G1 rescue mice. Approximately 50% of the rescue mice died by 6 weeks of age, with no significant survival difference observed between females and males (p = 0.077), though females were underrepresented compared to males during the initial genotyping (28 females compared to 48 males, p = 0.022). C-D) Weight at genotyping (at 14–21 days) was on average 25–30% smaller for G1 rescues than their littermates (p = 7x10^-13^). E) Survival of rescue mice beyond G1 (≥G2) is also reduced, with worse outcome in females (p = 0.002). Across all pedigrees, mice beyond G1 (≥G2) continued to exhibit reduced survival with more pronounced underrepresentation of females (p = 0.002), and F) an average ~22% lower body weight compared to littermates (mean defined as 100%) at the time of genotyping (p = 2x10^-16^). (PDF) [file pgen.1007658.s001.pdf]

# Pedigree sizes

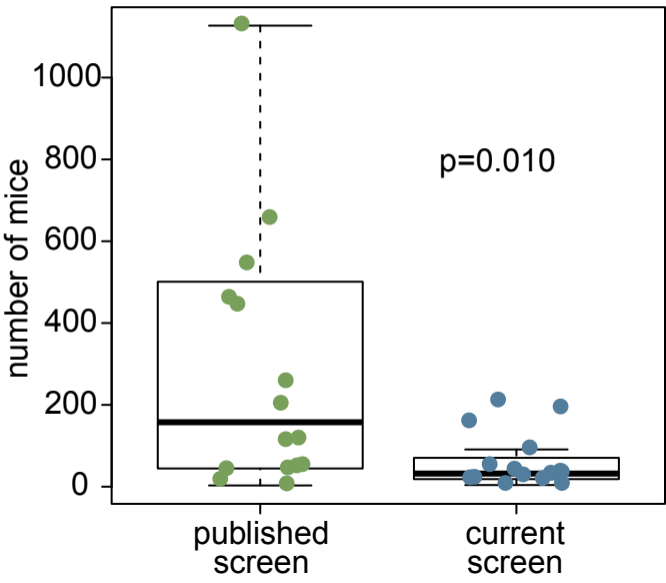

Supplement: S2 Fig — The ENU rescue pedigrees from the previous screen (n = 16, [5]) are significantly larger than the ENU rescue pedigrees observed in the current screen (p = 0.010, n = 15, S1 Table). (PDF) [file pgen.1007658.s002.pdf]

A

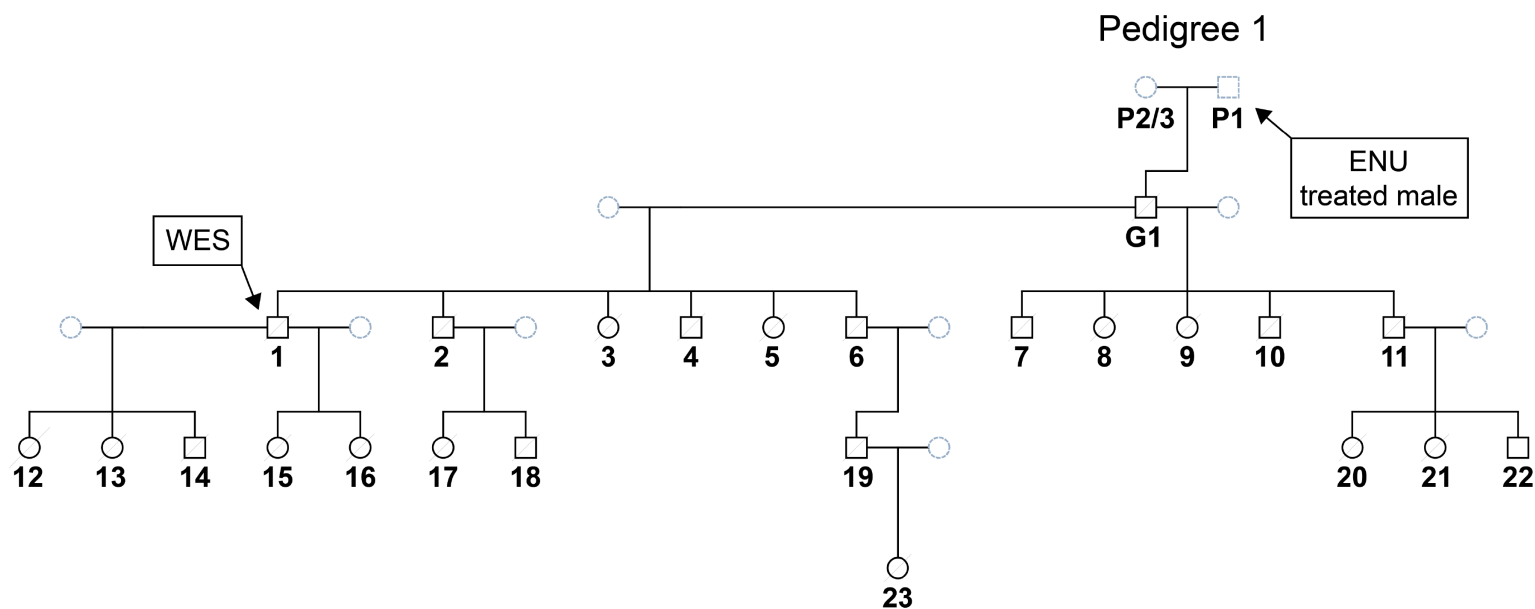

B

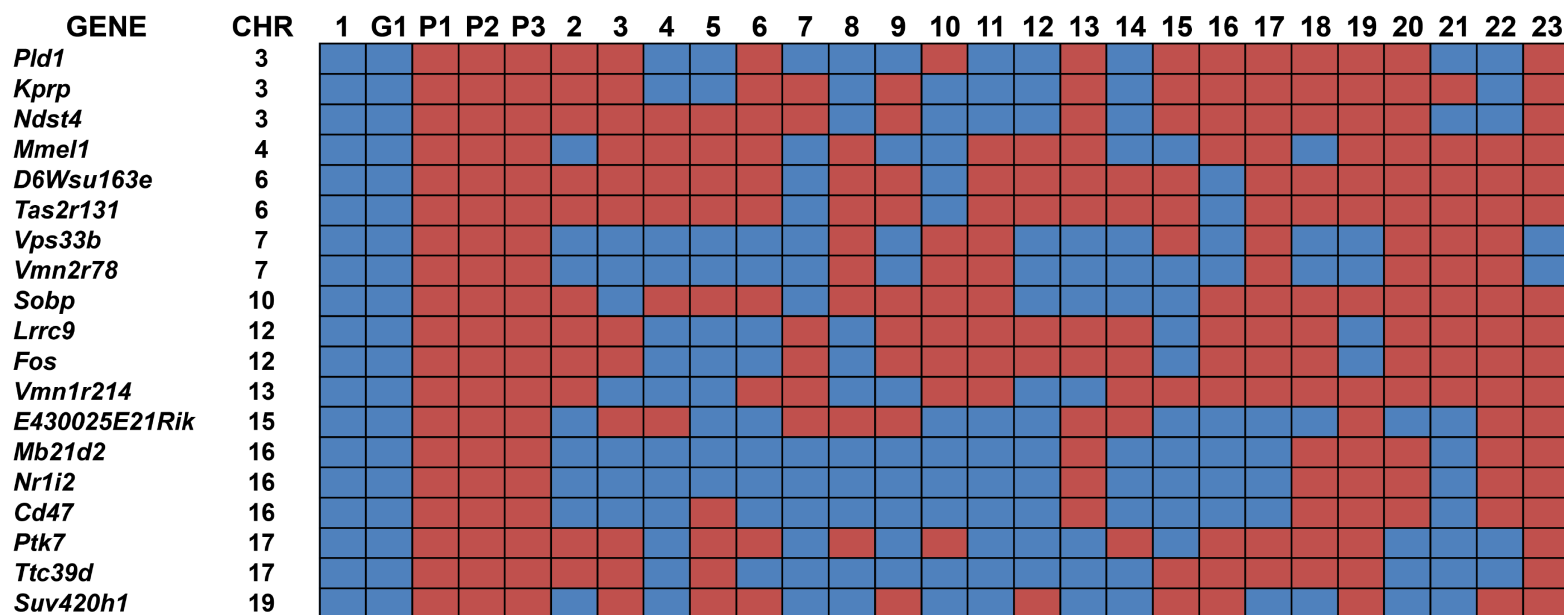

C

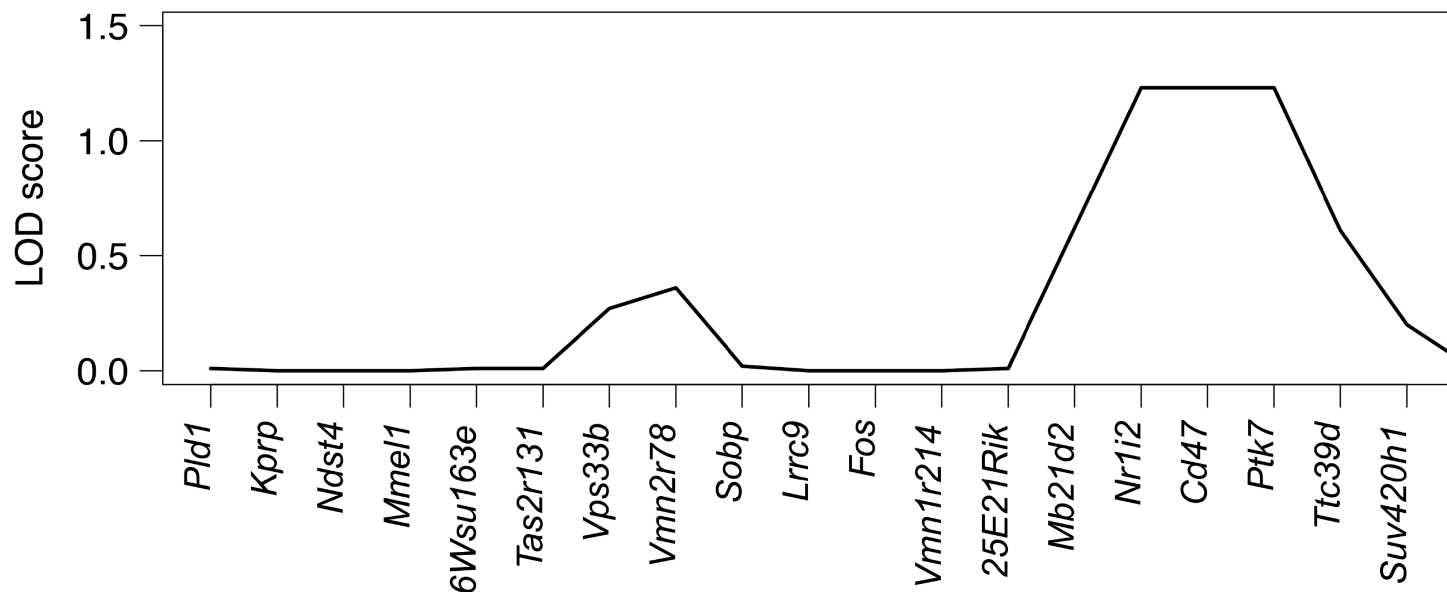

Supplement: S3 Fig — A) Overview of pedigree 1 (only rescue mice displayed). B) All coding ENU-induced mutations identified by WES were genotyped in all rescues from the pedigree by Sanger sequencing. Blue boxes indicate presence and red boxes indicate absence of the mutation. P1-P3 refers to 3 parental genotypes (G0 male and 2 untreated females). C) Linkage analysis using the ENU-induced variants from (B) as genetic markers. (PDF) [file pgen.1007658.s003.pdf]

A

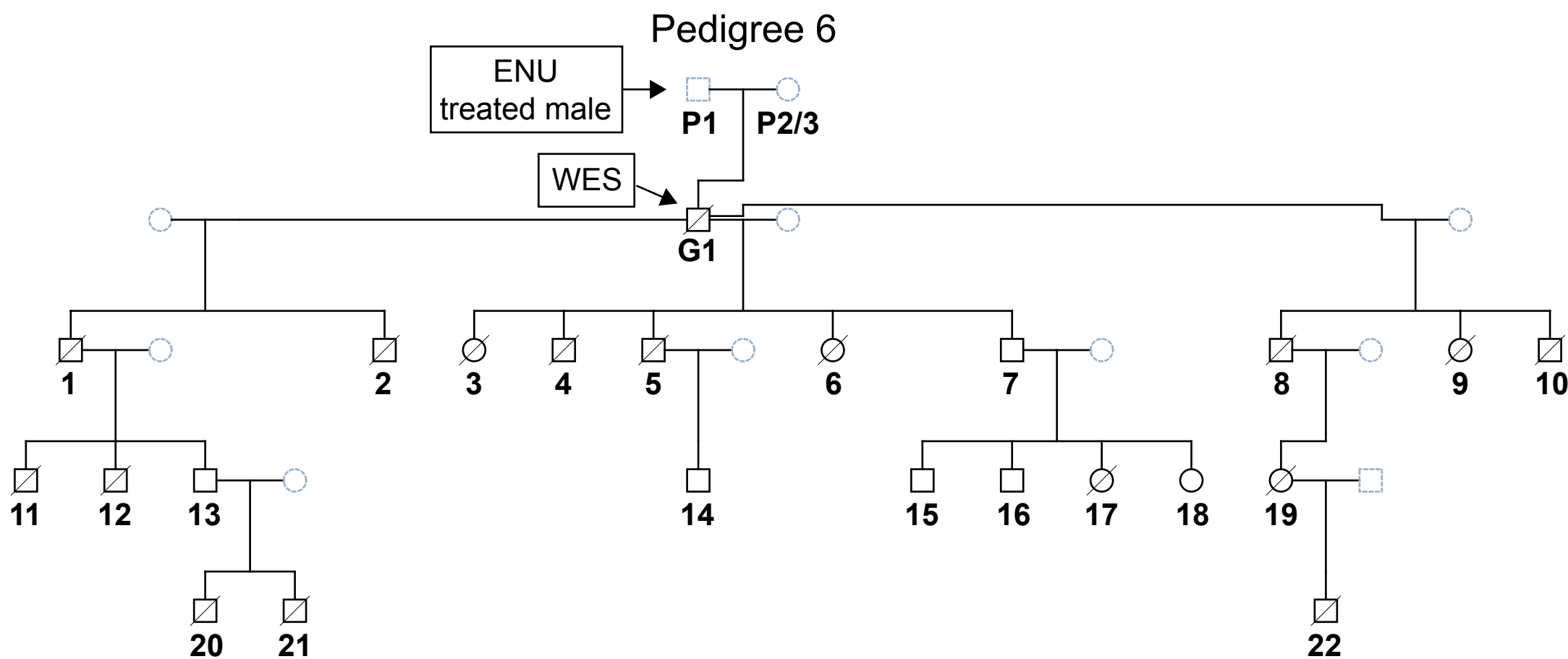

B

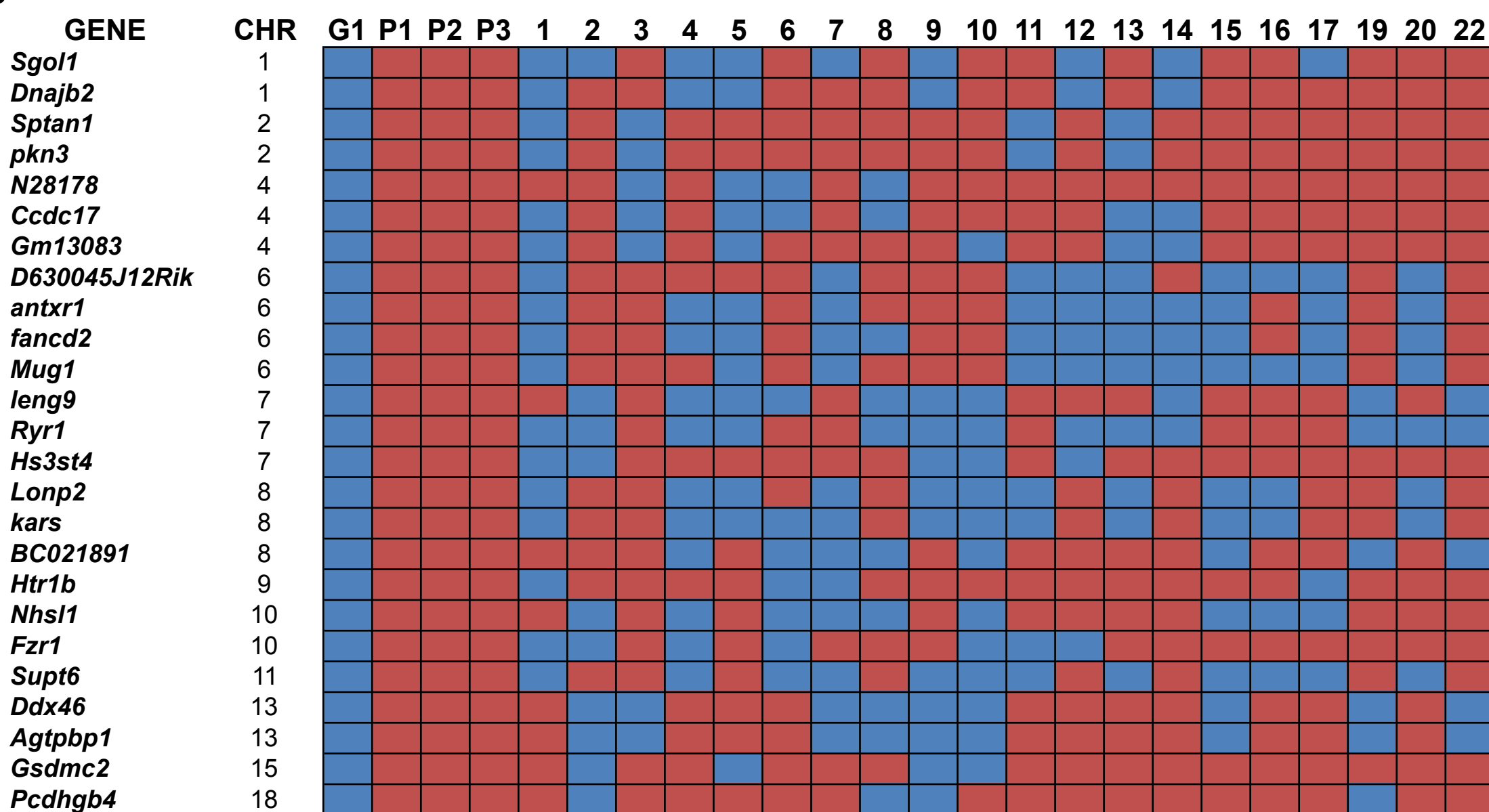

C

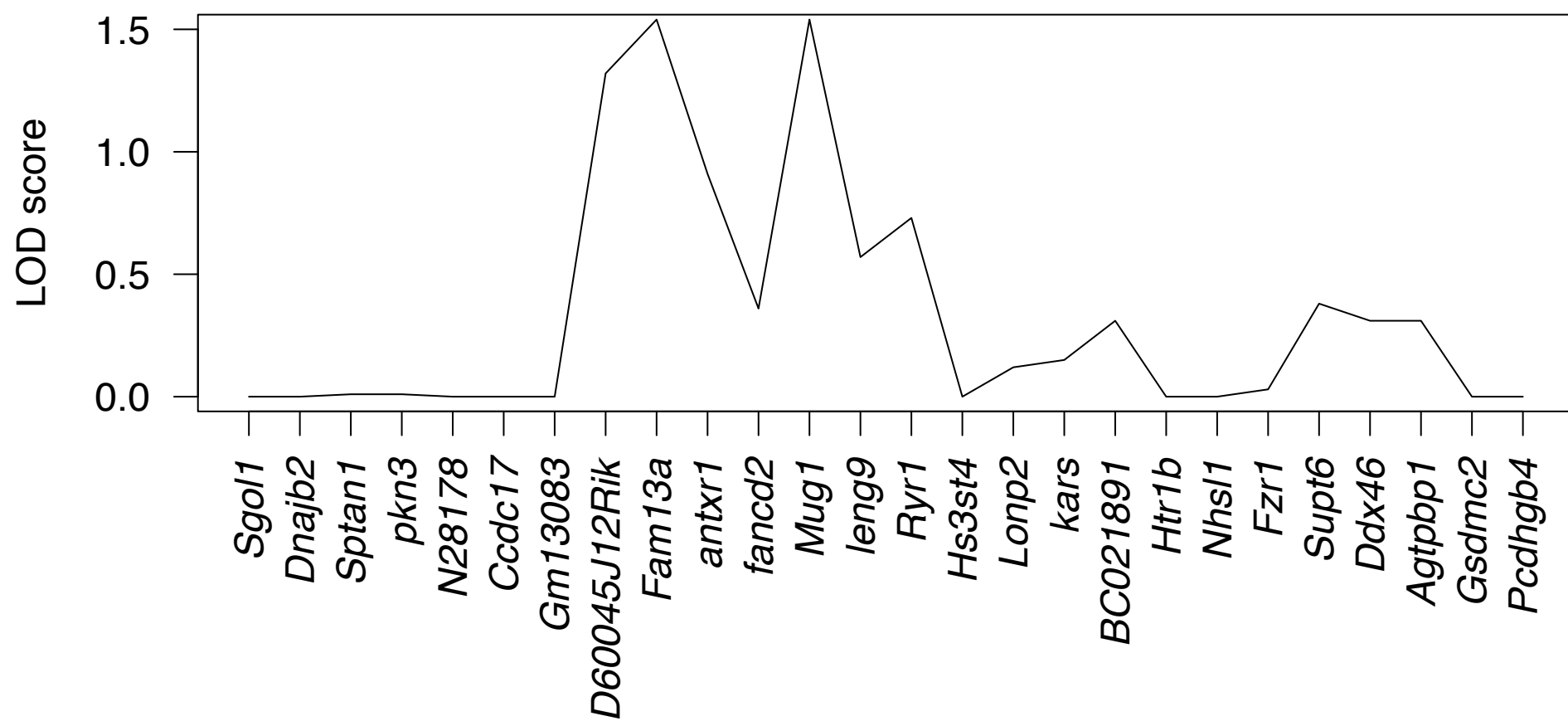

Supplement: S4 Fig — A) Overview of pedigree 6 (only rescue mice displayed). B) All coding ENU-induced mutations identified by WES were genotyped in most rescues from the pedigree by Sanger sequencing. Blue boxes indicate presence and red boxes indicate absence of the mutation. P1-P3 refers to 3 parental genotypes (G0 male and 2 untreated females). C) Linkage analysis using the ENU-induced variants from (B) as genetic markers. (PDF) [file pgen.1007658.s004.pdf]

A

Pedigree 13

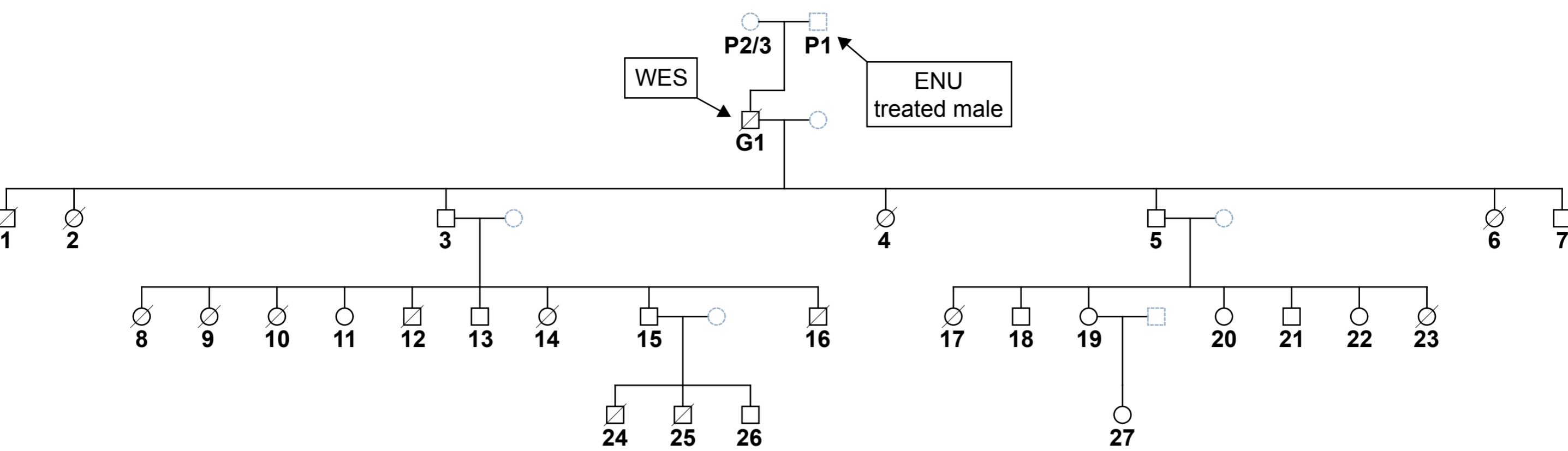

B

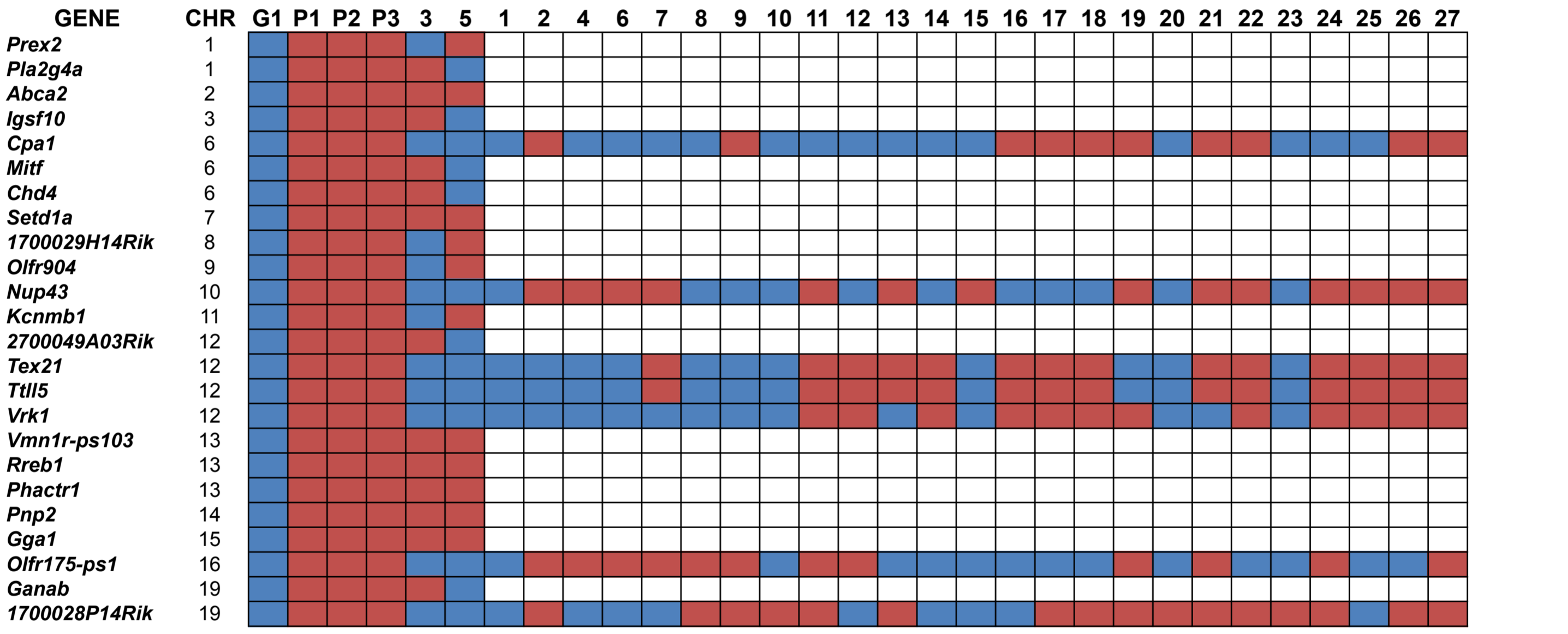

C

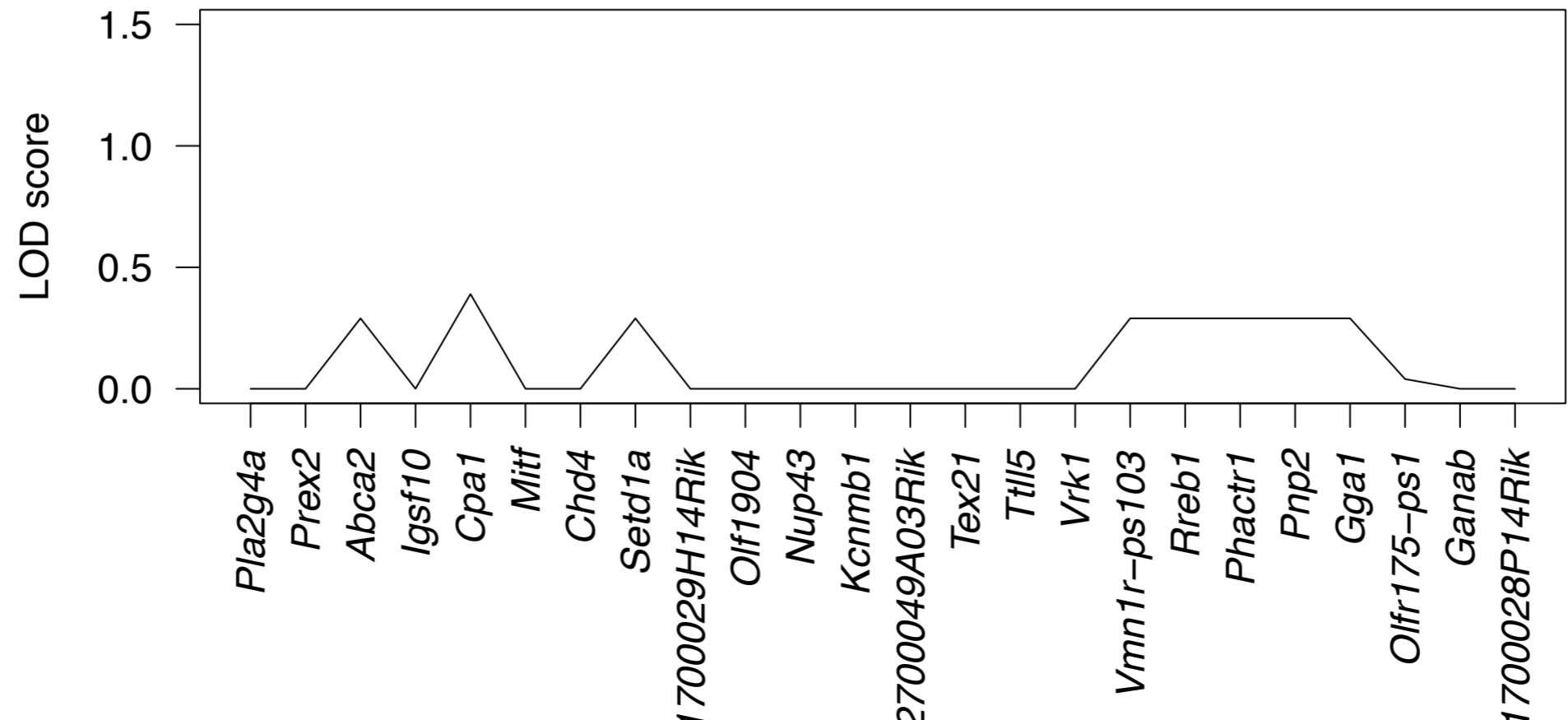

Supplement: S5 Fig — A) Overview of pedigree 13 (only rescue mice displayed). B) All coding ENU-induced mutations identified by WES were genotyped in all rescues from the pedigree if present in key mice 3 and 5 by Sanger sequencing. Blue boxes indicate presence and red boxes indicate absence of the mutation. P1-P3 refers to 3 parental genotypes (G0 male and 2 untreated females). C) Linkage analysis using the ENU-induced variants from (B) as genetic markers. (PDF) [file pgen.1007658.s005.pdf]

A

Segregation for *Pyhin1* in Pedigree 13

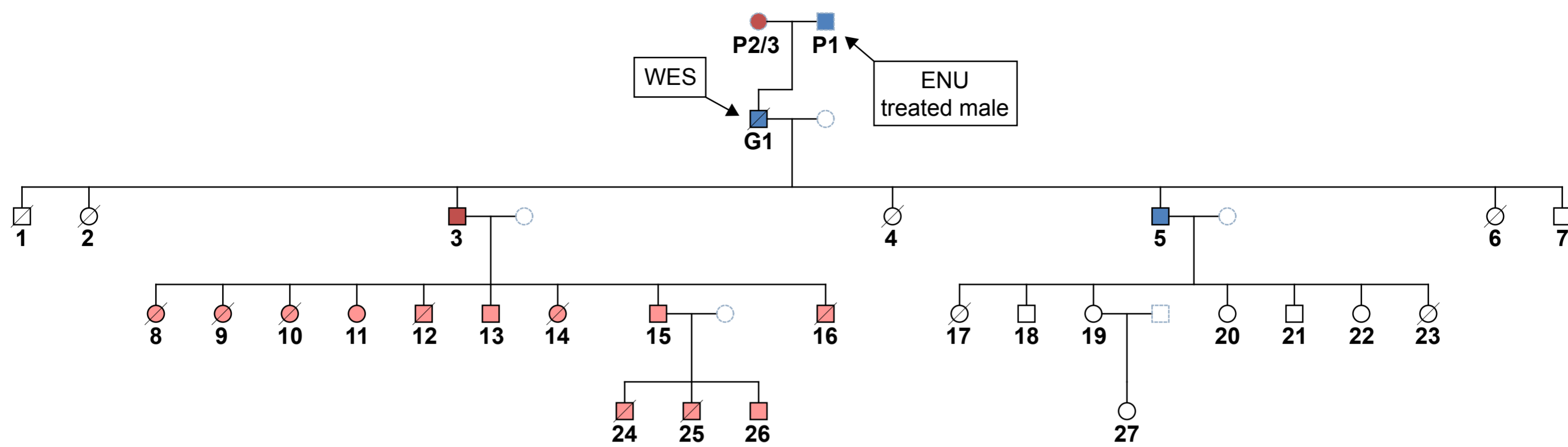

B

Segregation for *Fignl2* in Pedigree 13

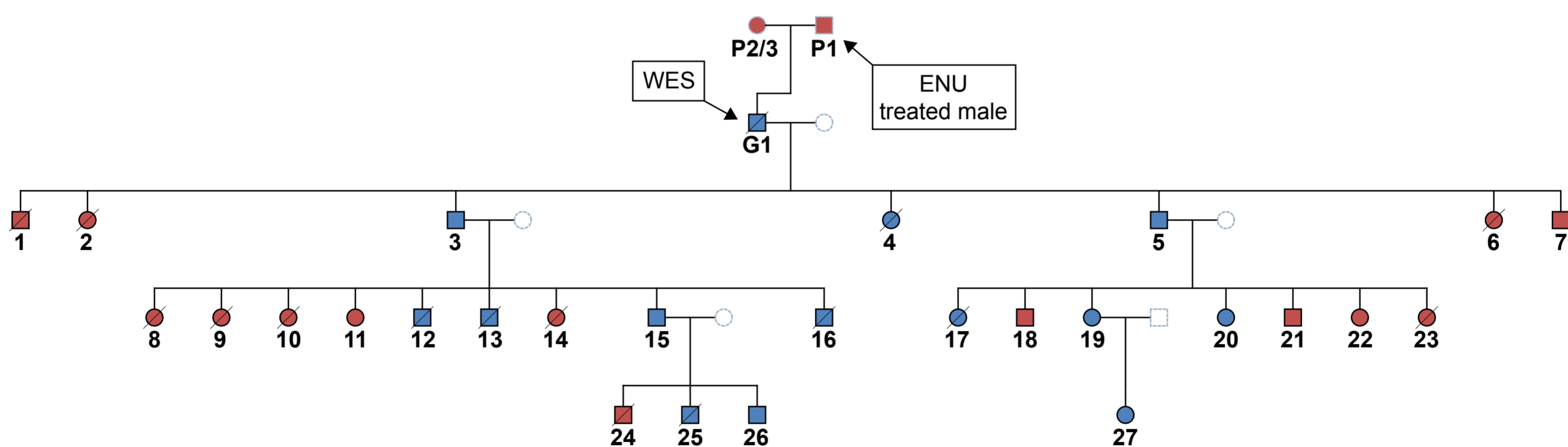

Supplement: S6 Fig — Segregation analysis in pedigree 13 for A) Pyhin1 and B) Fignl2 variants. Blue boxes indicate presence and red boxes indicate absence of the mutation. White boxes indicate untested mice, while light red boxes indicate untested mice with assumed absence of the mutation. (PDF) [file pgen.1007658.s006.pdf]

A

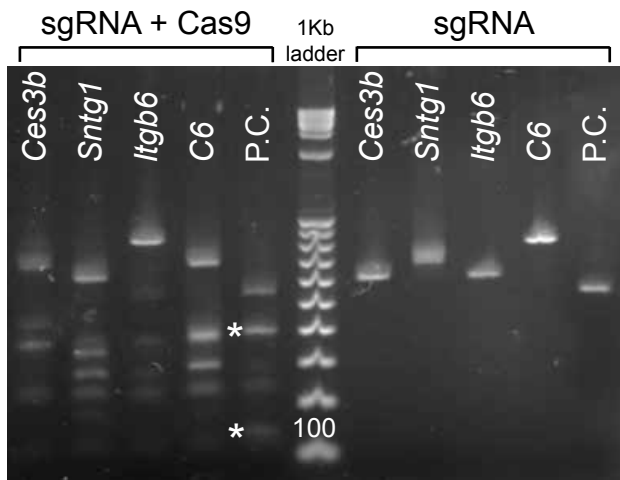

B

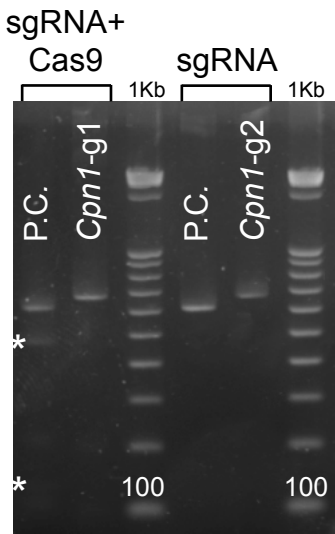

Supplement: S7 Fig — A) sgRNA+Cas9 targeting created double strand breaks in DNA templates obtained from genomic DNA by PCR. Expected sizes after sgRNA+Cas9 endonuclease activity: 430bp/240bp (Ces3b), 334bp/273bp (Sntg1), 530bp/275bp (Itgb6), and 383bp/296bp (C6). B) sgRNA+Cas9 complexes targeting Cpn1 using two different guides (g1, g2) failed to generate detectable double strand breaks. Positive control (P.C.) was added to ensure Cas9 protein activity, with expected sizes after cleavage (390bp/140bp) indicated by white stars. (PDF) [file pgen.1007658.s007.pdf]
